# Digital Hazards for Feeding and Eating - meta-analysis and discussion of putative mechanisms

**DOI:** 10.1192/j.eurpsy.2022.400

**Published:** 2022-09-01

**Authors:** K. Ioannidis

**Affiliations:** Cambridge and Peterborough NHS Foundation Trust, S3 Eating Disorders, Cambridge, United Kingdom

**Keywords:** meta-analysis, internet addiction, Eating Disorders, problematic use of the internet

## Abstract

**Introduction:**

Eating disorders are widespread illnesses with significant impact. There is growing concern about how those at risk of eating disorders overuse online resources to their detriment.

**Objectives:**

We present systematically gathered and pooled quantitative evidence from our review and meta-analysis study which aimed to provide a quantitative synthesis of all available data linking problematic usage of the internet (PIU) and eating disorder and related psychopathology. We synthesize how PUI influences eating disorder and related psychopathology, and examine what the moderating parameters influencing this relationship are.

**Methods:**

Our systematic review and meta-analysis protocol was pre-registered electronically in PROSPERO international register and included case-control studies using correlational statistics of association between internet use (various facets) and eating disorder psychopathology. Experimental and prospective studies are systematically reviewed separately.

**Results:**

The meta-analysis comprised n=32,295 participants, in which PUI was correlated with significant eating disorder general psychopathology Pearson r=0.22 (s.e.=0.04, p<0.001), body dissatisfaction r=0.16 (s.e.=0.02, p<0.001), drive-for-thinness r=0.16 (s.e.=0.04, p<0.001) and dietary restraint r = 0.18 (s.e.=0.03). Effects were not moderated by gender, PUI facet or study quality. Results are in support of PUI impacting on eating disorder symptoms; males may be equally vulnerable to these potential effects. Prospective and experimental studies in the field suggest that small but significant effects exist and may have accumulative influence over time and across all age groups.

**Conclusions:**

Those findings are important to expand our understanding of PUI as a multifaceted concept and its impact on multiple levels of ascertainment of eating disorder and related psychopathology. Putative specific effects of PUI on EDs are discussed.

**Disclosure:**

No significant relationships.

